# Does social capital enhance political participation in older adults? Multi-level evidence from the European Quality of Life Survey

**DOI:** 10.1007/s10433-024-00825-x

**Published:** 2024-10-14

**Authors:** Fredrica Nyqvist, Rodrigo Serrat, Mikael Nygård, Marina Näsman

**Affiliations:** 1 Faculty of Education and Welfare Studies, Social Policy, Åbo Akademi University, Vaasa, Finland; 2 Department of Cognition, Development, and Educational Psychology, University of Barcelona, Barcelona, Spain

**Keywords:** Civic engagement, Political participation, Social capital, Older adults, Europe

## Abstract

This study explored the role of social capital for non-institutionalised and institutionalised political participation among older adults compared to younger age groups using European Quality of Life Survey data (EQLS) from 2016 and 33 countries (*n* = 36,908). Multilevel logistic regression analysis was employed to assess the association between individual- and country-level social capital and political participation. Findings revealed that, at the individual level, active associational engagement was positively related to both forms of political participation, while social trust and political trust were linked only to non-institutionalised participation—higher social trust and lower political trust were associated with a greater likelihood of participation. Country-level associational engagement was related to non-institutionalised political participation and country-level political trust with institutionalised participation. For older adults, active associational engagement had stronger individual and contextual effects on non-institutionalised political participation. These results enrich our understanding of political engagement in later life and highlight the unexplored potential for civic involvement among older adults.

## Introduction

In this article, we contribute to the literature by examining civic engagement, particularly political participation, in later life, and how this is related to social capital. This topic is important to investigate for two reasons: first, higher participation of older adults is linked to many favourable outcomes, such as improved well-being (Serrat et al. 2017), and second, it is a matter of democracy that older adults get their rights and have their voices heard (e.g. Goerres 2009). We examine older adults’ political participation in comparison with younger age groups, as well as the interplay between age and individual and country-level social capital on non-institutionalised and institutionalised political activities. While social capital is embedded and facilitated by societal factors, we find it relevant to add country-level social capital as a macro-level predictor of political participation, as well as a possible moderator between age and political participation. While the association between social capital and political participation has been the focus of many studies (e.g. La Due Lake and Huckfeldt 1998; Teney and Hanquinet 2012), the meaning of social capital for political participation among older adults has scarcely been addressed. Therefore, by analysing age group differences, this study provides more insight into whether the same social capital predictors affect political participation in older adults and younger age groups. In the following sections, we discuss political participation as one key aspect of civic engagement and the connection between social capital and political participation before providing an overview of the literature on age differences in social capital and political participation, with an emphasis on older adults.

### Two modes of political participation within civic engagement

Along with formal volunteering, political participation is one of the two key dimensions of civic engagement in later life. Civic engagement, understood as ‘older people’s unpaid, nonprofessional activities aimed at seeking improved benefits for others, the community or wider society, or impacting on collective decision-making processes’ (Serrat et al. 2022), should be recognised as a multidimensional concept comprising a wide range of activities. Some authors have proposed distinguishing between two fundamental dimensions within this concept: what has been termed volunteering (e.g. Serrat et al. 2022, p. 624), community participation (e.g. Adler and Goggin 2005) or pre-political or latent political participation (e.g. Ekman and Amnå 2012) and political participation itself (e.g. Adler and Goggin 2005; Ekman and Amnå 2012; Serrat et al. 2022). While the first dimension encompasses all activities aimed at helping others, the community or wider society and—in general—promoting the common good without manifest political objectives, the second pertains to activities explicitly directed at influencing political decision-making processes (Serrat et al. 2022).

Research on older people’s civic engagement has advanced significantly in recent decades. However, most studies have primarily concentrated on older people’s formal volunteering, leaving a notable gap in the research on political participation in later life. In fact, a scoping review published in 2020 found that while 83% of the 429 papers published on the topic of late-life civic engagement addressed formal volunteering, only slightly over 10% focused on older people’s political participation (Serrat et al. 2020). Political participation is not easier to define than civic engagement. Political participation has also been recognised as a multidimensional concept encompassing various types of activities and has been defined as ‘the individual, non-professional and voluntary participation in activities that aim, directly or indirectly, at influencing political outcomes, changing the institutional premises for politics or affecting the selection of personnel or their choices’ (Nygård and Jakobsson 2013a, pp. 67). While earlier definitions of the concept limited it to electoral participation, subsequent expansions incorporated non-electoral modes of participation, such as contacting representatives or engaging in political organisations, as well as expressions of dissent and rejection, such as protesting or participating in social movement organisations (Theocharis and van Deth 2018; van Deth 2015). Contemporary understandings of the concept distinguish between institutionalised and non-institutionalised political participation (e.g. Goerres 2009). The former refers to activities conducted through established and regulated political entities and processes. In contrast, the latter involves less formal grassroots forms of political participation that citizens may use to challenge the status quo (Serrat et al. 2021; Silagadze et al., 2023). Examples of the former include contacting politicians or participating in political organisations, while examples of the latter include signing petitions or boycotting products (Bäck and Christensen 2016; Hooghe and Marien 2012; Silagadze et al. 2023).

### Social capital and political participation

Starting from Putnam’s work in the early 1990s, research on social capital and political participation has attracted scholarly interest (e.g. Bäck 2011; La Due Lake and Huckfeldt 1998; Norris 2002; Putnam 1993). Theoretically, social capital offers an explanation as to why an active civil society is beneficial for a vibrant and healthy democracy. Social capital has been defined as ‘features of social organisation, such as trust, norms and networks, that can improve the efficiency of society by facilitating coordinated actions’ (Putnam 1993, p. 167). This implies that when people feel connected within the community and experience relatively high levels of trust, they are more likely to develop positive attitudes towards political processes and governance, fostering greater confidence in political participation.

In the literature, a frequent distinction has been drawn between structural components—such as networks formed through associational engagement—which connect individuals and groups, and cognitive components, which manifest the values, trust and confidence defining these relationships (Putnam 2000). While social trust refers to the confidence individuals have in one another, institutional or political trust pertains to trust in political institutions and governance systems (Putnam 2000). On the one hand, social trust between citizens is essential for social cohesion, integration and stability—features that are crucial for a well-functioning society. On the other hand, if trust in various decision-making institutions (e.g. parliament) is high, people are more likely to live by the policies and rules of these institutions.

While classical conceptualisations of social capital have primarily emphasised social trust and social networks, political or institutional trust plays a similarly important role in facilitating coordinated actions within society (Gabriel 2017). Woolcock’s (2001) concept of linking social capital highlights the importance of connections between individuals or groups and institutions that hold power. In this framework, political trust plays a key role, as it reflects the confidence in these institutions, facilitating access to resources and opportunities. Therefore, political trust is significant in shaping the relationship between social capital and political participation. Besides defining social capital as an individual resource, it has also been shown that in societies, regions or even welfare regimes, being rich in social capital reinforces cooperative attitudes and practices, thereby promoting collective civic action and governance (Putnam 2000). Thus, there is a contextual effect of social capital on political participation, in addition to an individual effect.

However, evidence on the relationship between different aspects of social capital and political participation has remained elusive. On an individual level, the positive association between associational engagement and political participation represents a well-established research finding (Verba et al. 1995). The civic voluntarism model proposed by Verba et al. (1995) points to the important socialising capacity of associations to improve the necessary civic skills and capacities when taking political action outside the associations’ sphere. Research conducted in Europe has shown the benefits of associational engagement in relation to the political participation of migrants (Giugni and Grasso 2020; Serrat et al. 2023), young people (Grasso and Smith 2022) and older adults (Nygård et al. 2015; Serrat et al. 2015).

The relationship between trust and political participation seems to be more complex (see e.g. Bäck and Christensen 2016; Levi and Stoker 2000). Some studies point to a positive association between social trust and political participation (Putnam 2000), whereas others report a non-significant relationship (e.g. Uslaner and Brown 2005). A tentative explanation for the diverging results might be related to whether institutionalised or non-institutionalised political participation is under study (Norris 2002). While trust may compel citizens to engage in institutionalised forms of political participation, distrust, on the contrary, may be associated with elite-challenging, non-institutionalised expressions of dissent against the political system (Hooghe and Marien 2012; Bäck and Christensen 2016). Another explanation involves the fact that the relationship between social trust and participation is context-bound such that aggregated trust might have an important role in explaining the association on an individual level (Marien and Christensen 2013). It seems like social trust is more connected to non-institutionalised political participation (Uslaner and Brown 2005), although an association with institutionalised participation has also been reported (Bäck and Christensen 2016). Previous studies have acknowledged that individuals with high political trust are more inclined to engage in institutionalised political participation, whereas low political trust can be a motivating factor for participation in non-institutionalised political activities, such as signing petitions or demonstrating (Hooghe and Marien 2012; Mattila 2020). Political trust is also clearly context bound and is connected to political culture, historical traditions and economic development of the country (Zmerli and Hooghe 2011).

Based on the theoretical discussion on social capital and political participation, we can outline two different hypotheses.

*Individual-level hypothesis* (H1): Social capital is a significant predictor of political participation at the individual level. Specifically, while associational engagement positively influences both non-institutionalised and institutionalised forms of political participation, the effects of political and social trust vary depending on the form of political participation. *Country-level hypothesis* (H2): Political participation is influenced by country characteristics, particularly country-level social capital. Higher levels of social capital at the national level are associated with higher political participation.

### Older adults’ political participation

Studies on older adults’ political participation have been limited and have primarily focused on institutionalised political activities (e.g. Serrat et al. 2015; Wenner and Wagner 2022), with less evidence available regarding older people’s participation in non-institutionalised political activities or comparisons between both types of political participation (e.g. Nygård and Jakobsson 2013a, 2013b). Evidence of the impact of social capital on older people’s political participation is even more scarce. In general, research indicates a positive correlation between associational engagement and institutionalised and non-institutionalised political activities, while findings regarding variables such as marital status or employment status remain inconclusive (e.g. Boerio et al. 2023; Purdam and Taylor 2023; Serrat et al. 2023).

Very few studies have examined the role of trust in late-life political participation, demonstrating positive associations of political trust (e.g. Nygård and Jakobsson 2013a) or institutional trust (e.g. Nygård and Jakobsson 2013b) with institutionalised political activities but no association with non-institutionalised political activities. Regarding social trust, Nygård and Jakobsson (2013b) found a positive association with voting but no association with other forms of institutionalised political participation, such as contacting politicians. Furthermore, no association was found with non-institutionalised forms of political participation.

Age is a significant factor in understanding political participation, with the relationship between age and political participation described as curvilinear—both younger and older people tend to participate less than the middle-aged (e.g. Solevid and Scheiber Gyllenspetz 2022; Bui et al. 2023; Tambe and Kopacheva 2023). The evidence also suggests that older people are more likely to be engaged in institutionalised activities compared to younger age groups, but less likely to be engaged in non-institutionalised activities (Melo and Stockemer 2014). There are different explanations as to why age, particular with a focus on older adults, influences political participation. Specifically, older individuals, especially those who are middle aged and older, possess adequate time and resources, which are conducive to political engagement (e.g. Verba et al. 1995). Additionally, the socialisation mechanisms within families, which are crucial for encouraging political engagement among younger individuals (Verba et al. 2005), have seemingly diminished in significance regarding the acquisition of political information and fostering engagement.

Age differences could also be related to life course experiences and individual changes throughout life, such as transitions from education to working life to retirement (Weiss 2020). Additionally, a decline in social- and health-related resources in later life may negatively affect civic engagement, including political participation (Serrat et al. 2020). Moreover, older adults’ political participation may stem from cohort-related institutional and societal factors, such as the stability and vitality of the democracy during an individual's political socialisation (Inglehart and Welzel 2005), as well as the increasing dependency of older adults on the state through various welfare benefits, which could mobilise strong interest groups in later life (Campbell 2003).

The latter point implies that broader societal dynamics, including country-level social capital, may shape age-related differences in political engagement. For example, in countries with robust social capital, norms that encourage political involvement are reinforced (Putnam 2000), potentially moderating any negative impact of age on political participation. Moreover, strong social capital fosters inclusive societies (Liamputtong et al. 2022), empowering individuals of all ages within political processes. Countries with substantial social capital typically exhibit stronger social networks and higher levels of trust (Pichler and Wallace 2007). It is plausible that older adults benefit from these networks and trust, enhancing their engagement despite age-related changes, such as declining health. To our knowledge, no study has compared age differences in social capital in relation to non-institutionalised and institutionalised political participation, specifically considering that older adults might benefit particularly from living in a cohesive society. Given the dynamics of age on political participation and the influence on country-level social capital and political participation the final hypothesis that will be tested in the empirical section is:

#### Hypothesis (H3)

Country-level social capital moderates the effect of age on political participation. In countries with stronger social capital, older adults are more likely to engage in both non-institutionalised and institutionalised political activities compared to countries with weaker social capital.

To sum up, the empirical evidence on the relationship between social capital and non-institutionalised and institutionalised political participation in older adults is scant (e.g. Nygård and Jakobsson 2013a, 2013b; Serrat et al. 2023). The few existing studies are mostly limited to specific geographical contexts and do not cover a wide array of European countries. We also lack an age-comparative viewpoint to help us understand the role of social capital in political participation in old age. Drawing from previous literature (e.g. Nygård et al. 2015; Nygård and Jakobsson 2013a; Serrat et al. 2023), it is evident that political participation is influenced by various factors, including marital status and socio-structural resources, such as health and socioeconomic status. These factors were incorporated into our study as potential confounding variables. The overall aim of this study is, therefore, to test the importance of individual- and country-level social capital to non-institutionalised and institutionalised political participation in older adults compared to younger age groups by analysing cross-national data from the European Quality of Life Survey (EQLS) in 2016. Specifically, we assess (O1) country differences in political participation in younger and older adults, (O2) the association between social capital and political participation and (O3) age differences in the association between social capital and political participation.

## Data and methods

### Data source

The analyses are based on the fourth and most recent wave of the EQLS conducted in 2016 by Eurofound. The survey included individuals aged 18 and over in the following 33 European countries: Albania, Austria, Belgium, Bulgaria, Cyprus, Croatia, the Czech Republic, Denmark, Estonia, Germany, Greece, Finland, France, Hungary, Ireland, Italy, Lithuania, Luxembourg, Latvia, Malta, Montenegro, the Netherlands, North Macedonia, Poland, Portugal, Romania, Serbia, Slovenia, Slovakia, Spain, Sweden, Turkey and the United Kingdom. The survey was conducted using computer-assisted personal interviewing (CAPI). The achieved sample included in EQLS was 36,908 individuals aged 18 and over from all 33 countries. The number of individuals per country ranged from 1000 in Latvia and Malta to 2,019 in Turkey. The total response rate was 37%, and the highest and lowest response rates were observed in Montenegro (69%) and Sweden (16%), respectively. More information about the data collection can be found in the technical and fieldwork report (Eurofound 2018). The EQLS incorporates various indicators of social capital and political participation, including issues that are less explored in some of the larger cross-national European datasets. This allows for a more comprehensive examination of political participation across European countries. The percentage of respondents with missing data ranged from 0.1 to 1.5% for the variables included in the analysis.

### Measures

#### Dependent variables

Two different aspects of political participation were included in the analyses: non-institutionalised and institutionalised political participation. In line with previous literature (e.g. Marien et al. 2010), non-institutionalised political participation was operationalised by combining several indicators of informal character: (a) attended a protest or demonstration, (b) signed a petition, (c) commented on a political or social issue online or (d) boycotted certain products. An individual was considered to have participated in non-institutionalised political activities if they stated they had done any of these activities during the last 12 months (= 1).

Institutionalised political participation was operationalised by combining three indicators of formal character: (a) done voluntary work for political parties, trade unions, social movements or charities, (b) contacted a politician or public official, or (c) attended a meeting of a trade union, a political party or political action group. Similar to non-institutionalised political participation, a person was considered politically active if they stated they had done either one of these activities during the last 12 months (= 1). An exploratory factor analysis validated these two distinct dimensions of political participation (see Appendix 1).

#### Independent variables

This study used three social capital variables as key independent variables. *Associational engagement* was based on how frequently the respondent participates in the social activities of a club, society or association. A person was considered to participate in an association if they chose the option every day or almost every day, at least once a week or one to three times a month (= 1), as opposed to less often or never (= 0). *Social trust* was measured using the following question: ‘Generally speaking, would you say that most people can be trusted or that you can’t be too careful in dealing with people? Please tell me on a scale of 1 to 10, where 1 means that you can’t be too careful, and 10 means that most people can be trusted.’ *Political trust* was measured by respondents’ trust in the national parliament on a 10-point Likert scale, from (1) do not trust at all to (10) trust completely. The average of social and political trust as well as the share of people active in associations (every day or almost every day, at least once a week or one to three times a month) at the country level provides the contextual measures of social capital.

The covariates in the regression analyses included age groups (18–34, 35–49, 50–64, 65–74, and 75 +), gender (male = 0; female = 1), educational level (based on ISCED-1997 codes: low secondary education or lower (ISCED score 0–2); upper secondary or post-secondary (ISCED score 3–4); tertiary (ISCED score 5–8); urbanisation (the open countryside, a village/small town = 0; a medium to large town, a city or city suburb = 1) and employment status (employed, retired, other (unemployed/unable to work due to illness/homemaker/student/other)). Two variables were used to assess marital and cohabiting status: has partner in the household (yes, no) and a question about current marital status (never married, married, separated, widowed, divorced). These two variables were grouped together and recoded into ‘married or living with partner’, ‘divorced or separated’, ‘widowed’ and ‘unmarried’. Self-rated health was assessed with the question, ‘In general, how is your health?’ Responses were based on a five-point scale (very good, good, fair, bad, very bad) and were grouped into ‘good health’ (very good/good = 1) and ‘poor health’ (fair/bad/very bad = 0). The adequacy of a respondent’s income was captured with the question, ‘Thinking of your household’s total monthly income, is your household able to make ends meet?’ The ability to make ends meet was recoded into ‘with difficulty’ (with some difficulty, with difficulty, with great difficulty = 0) and ‘without difficulty’ (very easily, easily, fairly easily = 1).

#### Analyses

Information on non-institutionalised and institutionalised political participation and social capital was first extracted as percentages and means according to country. To get a better overview, the ratios of political participation and social capital were first presented for two broad age categories: those aged 65 and over and those aged 18–64 years (Table 1). Then, multi-level logistic regression analysis was applied to account for the nested structure of the data, with individuals (at level 1) nested within European countries (at level 2). Missing values were handled through listwise deletion, as in logistic regression.

**Table 1:** Levels of non-institutionalised and institutionalised political participation, associational engagement, social and political trust among people < 65 and >  = 65 in 33 European countries, European Quality of Life Survey (EQLS) 2016

Country	Non-institutionalised political participation (%)		Ratio	Institutionalised political participation (%)		Ratio	Associational engagement (%)		Ratio	Social trust (mean)		Ratio	Political trust (mean)		Ratio
	< 65	≥ 65	≥ 65/ < 65	< 65	≥ 65	≥ 65/ < 65	< 65	≥ 65	≥ 65/ < 65	< 65	≥ 65	≥ 65/ < 65	< 65	≥ 65	≥ 65/ < 65
*North*															
Denmark	50.2	29.5	0.6	24.4	20.0	0.8	53.0	60.0	1.1	7.3	7.2	1.0	6.0	6.7	1.1
Finland	54.8	30.0	0.5	23.8	15.6	0.7	46.0	48.4	1.1	7.4	7.4	1.0	6.4	6.8	1.1
Netherlands	43.7	27.6	0.6	12.4	10.4	0.8	47.4	48.7	1.0	6.3	5.9	0.9	5.7	5.6	1.0
Sweden	72.1	54.8	0.8	35.1	24.0	0.7	54.4	64.5	1.2	6.8	6.2	0.9	6.4	6.5	1.0
*West*															
Austria	43.8	24.5	0.6	24.5	16.0	0.7	44.3	42.5	1.0	5.4	5.2	1.0	5.6	5.6	1.0
Belgium	43.2	22.5	0.5	21.1	10.6	0.5	40.1	34.6	0.9	5.4	5.1	1.0	5.0	5.1	1.0
France	44.2	30.0	0.7	18.5	13.9	0.8	26.6	35.9	1.3	5.4	5.5	1.0	4.4	4.5	1.0
Germany	38.3	29.1	0.8	20.3	20.5	1.0	42.7	46.0	1.1	5.2	4.7	0.9	5.4	5.7	1.0
Ireland	30.4	13.6	0.4	24.0	13.3	0.6	47.0	44.4	0.9	6.0	5.9	1.0	4.6	5.0	1.1
Luxembourg	46.4	33.3	0.7	17.9	16.7	0.9	35.7	16.7	0.5	5.9	5.8	1.0	5.9	6.3	1.1
United Kingdom	46.3	32.5	0.7	16.7	15.2	0.9	37.6	48.8	1.3	5.3	5.5	1.0	4.8	5.1	1.1
*South*															
Italy	32.9	9.7	0.3	13.9	11.1	0.8	20.7	19.2	0.9	5.2	5.2	1.0	3.7	3.9	1.1
Greece	28.2	10.5	0.4	6.8	4.1	0.6	13.2	4.7	0.4	4.1	4.0	1.0	2.7	3.2	1.2
Malta	23.8	16.7	0.7	9.5	16.7	1.8	19.0	16.7	0.9	5.0	5.0	1.0	4.9	5.6	1.1
Portugal	22.5	6.1	0.3	10.5	5.6	0.5	28.5	13.4	0.5	4.8	4.4	0.9	4.2	4.2	1.0
Spain	22.9	3.4	0.1	9.7	4.2	0.4	20.0	15.8	0.8	5.3	5.0	0.9	3.7	4.3	1.2
*East*															
Albania	9.2	3.8	0.4	11.1	7.4	0.7	14.1	3.8	0.3	2.4	2.7	1.1	2.5	3.4	1.4
Bulgaria	12.9	2.7	0.2	7.5	5.3	0.7	6.9	4.4	0.6	4.1	3.9	1.0	3.3	2.8	0.9
Croatia	22.3	14.8	0.7	11.7	8.2	0.7	28.6	10.2	0.4	3.8	3.7	1.0	3.1	3.6	1.2
Cyprus	16.7	10.0	0.6	11.6	10.0	0.9	20.9	11.1	0.5	3.1	2.7	0.9	3.6	4.1	1.1
Czech Republic	25.0	16.7	0.7	7.2	6.0	0.8	27.8	19.3	0.7	4.4	4.0	0.9	4.2	4.0	1.0
Estonia	23.4	5.6	0.2	7.8	6.0	0.8	35.9	11.1	0.3	5.0	4.9	1.0	4.8	5.0	1.0
Hungary	9.0	4.3	0.5	6.6	3.5	0.5	12.4	9.1	0.7	4.9	4.9	1.0	4.9	5.5	1.1
Lithuania	20.0	7.1	0.4	9.2	8.0	0.9	20.4	9.5	0.5	4.5	4.8	1.1	3.7	4.5	1.2
Latvia	17.7	3.3	0.2	9.4	6.5	0.7	18.9	12.9	0.7	4.5	4.3	0.9	3.6	4.1	1.1
Montenegro	20.0	14.3	0.7	19.4	14.3	0.7	30.0	14.3	0.5	4.6	4.2	0.9	5.3	5.4	1.0
North Macedonia	18.9	9.5	0.5	19.8	9.5	0.5	13.2	5.0	0.4	3.0	3.1	1.1	3.2	4.1	1.3
Poland	11.7	3.7	0.3	9.1	6.4	0.7	18.3	8.0	0.4	4.7	4.8	1.0	4.0	4.5	1.1
Romania	11.4	0.8	0.1	8.0	4.7	0.6	6.4	3.1	0.5	4.7	5.1	1.1	3.7	3.9	1.0
Serbia	25.3	7.0	0.3	24.5	12.5	0.5	29.6	10.8	0.4	4.5	4.3	1.0	4.6	5.0	1.1
Slovenia	21.8	10.3	0.5	10.8	6.9	0.6	37.6	24.1	0.6	4.8	4.6	1.0	3.2	3.1	1.0
Slovakia	20.0	5.0	0.3	8.6	1.7	0.2	22.6	15.0	0.7	4.0	4.1	1.0	3.8	5.1	1.3
Turkey	25.2	6.8	0.3	28.6	25.8	0.9	24.0	18.4	0.8	5.2	5.7	1.1	6.5	6.5	1.0

weighted by Wcalib_crossnational_total

First, the country-level variance in political participation was assessed without including any explanatory variables (empty model). Additionally, bivariate analyses were conducted with all included variables to explore their individual associations with political participation. Then, the relationship between individual social capital and political participation was examined, adjusting for other individual-level covariates (model 1). Subsequently, within-level interaction terms between individual-level social capital and age groups were included (model 2). The interaction effects were tested for the two political participation outcomes while also including all other variables.

Next, individual- and country-level social capital variables were modelled simultaneously (model 3). Finally, cross-level interaction terms between country-level social capital and age groups were added (model 4). In models 3 and 4, individual-level social capital was centred around the country mean to address collinearity issues between individual- and country-level social capital indicators. This centring technique helps to separate the individual-level effects of social capital on political participation from the contextual effects at the country level, making it possible to distinguish between within-country (individual) and between-country (contextual) variations in social capital (Enders and Tofighi 2007).

Finally, cross-level interaction terms between aggregate social capital and age groups were included, adjusting for all other variables. The cross-level interactions were interpreted and illustrated with the help of margins plots. Additionally, average marginal effects were calculated to understand how the effects of country-level social capital vary across different age groups. All models were conducted separately for non-institutionalised and institutionalised political participation. To check whether there was a multicollinearity problem, the variance inflation factor (VIF) was estimated. VIF scores ranged between 1.02 and 1.13, with a mean of 1.10, indicating no concern for multicollinearity to bias the results. The analyses were conducted using the melogit command in STATA 18. The descriptive analyses were weighted using Wcalib_crossnational_total, and the regression analyses were weighted using Wcalib, as recommended by Eurofound (Eurofound 2018).

## Results

Eighteen per cent of the respondents reported that they participated in non-institutionalised political activities, compared to 13% that reported that they took part in institutionalised activities. Table 1 shows the distribution of political participation among the countries included in the study according to two broad age groups. There were important differences between the countries when it came to institutionalised and non-institutionalised political participation. Non-institutionalised activities were the most popular in Northern and Western Europe, especially among those under 65 years of age. The participation rate varied between 9% in Hungary and 72.1% in Sweden. Only 0.8% of older adults (65 +) in Romania had done at least one of the non-institutionalised activities in the last year, whereas the highest active older adult group was found in Sweden (54.8%). In all countries, the younger age group (18–64 years) was more active than those aged 65 and over.

A similar age pattern was generally observed for institutionalised political participation, i.e. the younger age group was more active than the older age group, with the exception of Malta and Germany, where those aged 65 and over were more active. The participation rate was less diverse across various European regions for institutionalised political participation. The highest rate of institutionalised participation among the younger age group was found in  Sweden (35.1.%), while the lowest rate was in Hungary (6.6%). The corresponding highest and lowest values for those aged 65 and over were 25.8% in Turkey and 1.7% in Slovakia. Table 1 also shows the distribution of social capital according to country and age group.

To examine our hypotheses (H1–3), several multilevel logistic regressions were performed. An empty model revealed significant differences between the countries. Intra-class correlation in the null model for non-institutionalised political participation was 0.14, compared to 0.09 in the model for institutionalised political participation (see Appendix 2 for results of the empty model and the bivariate analyses). Thus, the results indicate that the included European societies differed more strongly from one another with regard to non-institutionalised participation than institutionalised political participation.

Then, the relationship between individual social capital and the two forms of political participation was examined, adjusting for other individual-level covariates (see model 1 in Tables 2 and 3). The results for non-institutionalised political participation revealed that holding all factors constant, an individual with higher associational engagement (OR 2.04, 95% CI 1.62–2.57) and social trust (OR 1.1, 95% CI 1.06–1.14) was more likely to be active in non-institutionalised activities than an individual with lower levels of participation and trust. There was a negative association between political trust and non-institutionalised activities (OR 0.92, 95%, CI 0.89–0.95), implying that higher levels of political trust make an individual less likely to be politically active in non-institutionalised activities. The oldest age group (75 +) was less likely to be politically active (OR 0.41, 95% CI 0.27–0.60) than the youngest age group (18–34 years). The corresponding results for institutionalised political participation showed that those aged 35–64 were more likely to be active (35–49 years, OR: 1.34, 95% CI 1.12–1.61; 50–64 years, OR 1.53, 95% CI 1.25–1.88) than the youngest age group. In addition, of the social capital variables, individuals with higher associational engagement were more likely to be politically active in institutionalised activities (OR 3.02, 95% CI 2.44–4.06).

**Table 2:** Multilevel logistic models of testing factors associated with the probability to participate in non-institutionalised political participation

	M1			M2			M3			M4		
	OR	CI 95%	*p* value	OR	CI 95%	*p* value	OR	CI 95%	*p* value	OR	CI 95%	*p* value
Associational engagement	2.04	(1.62–2.57)	< .001	1.96	(1.55–2.47)	< .001	2.03	(1.61–2.56)	< .001	2.03	(1.61–2.55)	< .001
Social trust	1.1	(1.06–1.14)	< .001	1.06	(1.02–1.10)	0.001	1.10	(1.06–1.14)	< .001	1.10	(1.06–1.14)	< .001
Political trust	0.92	(0.89–0.95)	< .001	0.91	(0.85–0.97)	0.004	0.92	(0.89–0.95)	< .001	0.92	(0.89–0.95)	< .001
*Age groups* (ref = 18–34)												
35–49	1.09	(0.95–1.26)	0.221	0.75	(0.52–1.09)	0.125	1.09	(0.95–1.26)	0.224	0.56	(0.33–1.32)	0.185
50–64	1.06	(0.94–1.19)	0.338	0.71	(0.46–1.11)	0.137	1.06	(0.94–1.19)	0.343	0.71	(0.23–2.20)	.558
65–74	0.74	(0.48–1.16)	0.188	0.49	(0.23–1.07)	0.073	0.74	(0.48–1.16)	0.187	1.89	(0.72–5.00)	0.197
75 +	0.41	(0.27–0.60)	< .001	0.22	(0.10–1.07)	0.001	0.41	(0.27–0.60)	< .001	0.39	(0.17–0.91)	0.029
Gender (ref = male)	1.11	(0.99–1.26)	0.085	1.11	(0.98–1.25)	0.096	1.11	(0.98–1.25)	0.087	1.11	(0.98–1.25)	0.091
*Educational level* (ref = low secondary education or lower)												
Upper secondary or post-secondary	1.83	(1.47–2.27)	< .001	1.81	(1.45–2.26)	< .001	1.83	(1.47–2.27)	< .001	1.80	(1.45–2.24)	< .001
Tertiary	3.26	(2.25–4.72)	< .001	3.22	(2.23–4.64)	< .001	3.26	(2.25–4.72)	< .001	3.24	(2.27–4.62)	< .001
Level of urbanisation (ref = the open countryside. a village/small town)	1.26	(1.09–1.45)	0.002	1.26	(1.10–1.45)	0.001	1.26	(1.09–1.45)	0.002	1.26	(1.09–1.45)	0.001
*Employment status* (ref = employed)												
Retired	1.09	(0.89–1.32)	0.398	1.10	(0.89–1.35)	0.375	1.09	(0.89–1.33)	0.393	1.05	(0.88–1.27)	0.564
Other	1.01	(0.93–1.10)	0.754	1.02	(0.94–1.10)	0.692	1.01	(0.94–1.10)	0.732	1.01	(0.93–1.10)	0.858
*Marital and cohabiting status* (ref = married or living with partner)												
Divorced or separated	0.93	(0.77–1.12)	0.450	0.94	(0.77–1.13)	0.495	0.93	(0.77–1.12)	0.431	0.93	(0.77–1.14)	0.491
Widowed	0.71	(0.60–0.84)	< .001	0.71	(0.59–0.84)	< .001	0.71	(0.60–0.84)	< .001	0.72	(0.61–0.84)	< .001
Unmarried	1.21	(1.01–1.47)	0.043	1.22	(1.02–1.47)	0.030	1.21	(1.00–1.46)	0.045	1.22	(1.02–1.47)	0.032
Good self-rated health (ref = poor SRH)	1.10	(1.02–1.20)	0.016	1.09	(1.01–1.19)	0.033	1.11	(1.02–1.20)	0.013	1.09	(1.01–1.18)	0.025
Ability to make ends meet (ref = with difficulty)	0.86	(0.76–0.96)	0.010	0.86	(0.77–0.97)	0.012	0.85	(0.76–0.96)	0.008	0.85	(0.76–0.95)	0.005
**Within level interactions**												
Age groups*associational engagement												
35–49*associational engagement				1.03	(0.81–1.30)	0.823						
50–64*associational engagement				1.00	(0.79–1.25)	0.986						
65–74*associational engagement				1.17	(0.85–1.59)	0.337						
75 + *associational engagement				1.29	(0.79–2.11)	0.305						
Age groups*social trust												
35–49*social trust				1.05	(1.00–1.12)	0.063						
50–64*social trust				1.08	(1.04–1.12)	< .001						
65–74*social trust				1.01	(0.95–1.07)	0.804						
75 + *social trust				1.02	(0.96–1.08)	0.588						
Age groups*political trust												
35–49*political trust				1.02	(0.90–1.15)	0.793						
50–64*political trust				1.00	(0.93–1.07)	0.908						
65–74*political trust				1.06	(0.99–1.14)	0.095						
75 + *political trust				1.08	(0.96–1.21)	0.227						
Aggregate associational engagement							1.04	(1.03–1.06)	< .001	1.05	(1.03–1.06)	< .001
Aggregate social trust							1.13	(0.93–1.39)	0.224	1.12	(0.92–1.39)	0.261
Aggregate political trust							1.00	(0.80–1.23)	0.971	0.94	(0.77–1.16)	0.573
**Cross-level interaction**												
Age groups*aggregate associational engagement												
35–49*associational engagement										0.98	(0.98–0.99)	< .001
50–64*associational engagement										0.99	(0.98–1.00)	0.171
65–74*associational engagement										1.03	(1.02–1.05)	< .001
75 + *associational engagement										1.03	(1.01–1.05)	0.008
Age groups*aggregate social trust												
35–49*social trust										1.09	(0.90–1.32)	0.369
50–64*social trust										1.12	(0.83–1.51)	0.477
65–74*social trust										0.71	(0.52–0.97)	0.032
75 + *social trust										0.89	(0.59–1.32)	0.555
Age groups*aggregate political trust												
35–49*political trust										1.16	(1.06–1.26)	0.001
50–64*political trust										1.03	(0.90–1.78)	0.702
65–74*political trust										0.95	(0.78–1.16)	0.646
75 + *political trust										0.93	(0.67–1.28)	0.642
Constant	0.10	(0.06–0.17)	< .001	0.14	(0.07–0.26)	< .001	0.02	(0.01–0.04)	< .001	0.03	(0.01–0.07)	< .001
Intra-Class Correlation (ICC)	.1222529			.1212671		.039973			.0402842			
AIC	36,015.09			35,982.7		35,984.79		35,882.31				
BIC	36,184.18			36,244.79		36,179.25		36,152.85				
Log-pseudolikelihood	− 17,987.55		− 17,960.35		− 17,969.4			− 17,909.15				
Respondents (countries)	34,701 (33)		34,701 (33)		34,701 (33)		34,701 (33)					

*OR* Odds ratio, *CI* Confidence intervals

**Table 3:** Multilevel logistic models of testing factors associated with the probability to participate in institutionalised political activities

	M1			M2			M3			M4		
	OR	CI 95%	*p* value	OR	CI 95%	*p* value	OR	CI 95%	*p* value	OR	CI 95%	*p*-
value												
Associational engagement	3.02	(2.24–4.06)	< .001	2.40	(1.61–3.60)	< .001	3.01	(2.23–4.06)	< .001	3.01	(2.23–4.08)	< .001
Social trust	1.04	(1.00–1.08)	0.055	1.00	(0.95–1.06)	0.866	1.04	(1.00–1.08)	0.057	1.04	(1.00–1.08)	0.052
Political trust	1.03	(0.97–1.08)	0.351	1.04	(0.99–1.10)	0.128	1.03	(0.97–1.08)	0.370	1.02	(0.97–1.08)	0.376
*Age groups* (ref = 18–34)												
35–49	1.34	(1.12–1.61)	< .001	1.13	(0.66–1.94)	0.661	1.342	(1.12–1.60)	0.001	4.36	(1.68–11.30)	0.002
50–64	1.53	(1.25–1.88)	< .001	1.18	(0.71–1.95)	0.517	1.53	(1.25–1.88)	< .001	3.14	(1.32–7.45)	0.009
65–74	1.26	(0.90–1.76)	0.183	0.72	(0.36–1.43)	0.347	1.26	(0.90–1.76)	0.180	3.22	(1.02–10.17)	0.047
75 +	0.84	(0.49–1.44)	0.522	0.57	(0.27–1.21)	0.144	0.84	(0.49–1.43)	0.522	4.93	(1.15–21.08)	0.031
Gender (ref = male)	0.72	(0.66–0.79)	< .001	0.72	(0.66–0.78)	< .001	0.72	(0.66–0.79)	< .001	0.72	(0.66–0.79)	< .001
*Educational level* (ref = low secondary education or lower)												
Upper secondary or post-secondary	1.29	(1.04–1.61)	0.020	1.28	(1.02–1.59)	0.031	1.29	(1.04–1.61)	0.200	1.27	(0.91–1.45)	0.043
Tertiary	1.99	(1.36–2.89)	< .001	1.95	(1.33–2.86)	0.001	1.99	(1.37–2.89)	< .001	1.93	(1.01–2.36)	0.001
Level of urbanisation (ref = the open countryside, a village/small town)	1.00	(0.84–1.19)	0.981	0.98	(0.85–1.12)	0.755	0.98	(0.85–1.12)	0.717	0.98	(0.86–1.12)	0.777
*Employment status* (ref = employed)												
Retired	0.90	(0.70–1.15)	0.394	0.90	(0.70–1.17)	0.431	0.90	(0.70–1.15)	0.397	0.94	(0.71–1.26)	0.690
Other	0.75	(0.67–0.84)	< .001	0.76	(0.68–0.84)	< .001	0.75	(0.67–0.84)	< .001	0.75	(0.67–0.85)	< .001
*Marital and cohabiting status* (ref = married or living with partner)												
Divorced or separated	1.04	(0.87–1.24)	0.693	1.04	(0.89–1.29)	0.696	1.03	(0.86–1.24)	0.711	1.03	(0.86–1.22)	0.753
Widowed	0.70	(0.57–0.86)	0.001	0.71	(0.57–0.88)	0.002	0.70	(0.57–0.86)	0.001	0.71	(0.58–0.86)	< .001
Unmarried	1.00	(0.89–1.11)	0.945	1.01	(0.91–1.12)	0.849	1.00	(0.89–1.11)	0.939	1.00	(0.89–1.11)	0.948
Good self-rated health (ref = poor SRH)	0.86	(0.68–1.10)	0.237	0.85	(0.67–1.08)	0.193	0.87	(0.68–1.10)	0.241	0.86	(0.68–1.09)	0.211
Ability to make ends meet (ref = with difficulty)	1.01	(0.79–1.29)	0.933	1.01	(0.80–1.28)	0.924	1.01	(0.79–1.28)	0.949	1.01	(0.79–1.29)	0.939
**Within level interactions**												
Age groups*associational engagement												
35–49*associational engagement				1.29	(0.98–1.70)	0.073						
50–64*associational engagement				1.35	(0.99–1.85)	0.061						
65–74*associational engagement				1.29	(0.81–2.05)	0.289						
75 + *associational engagement				2.15	(1.65–2.80)	0.000						
Age groups*social trust												
35–49*social trust				1.03	(0.92–1.16)	0.576						
50–64*social trust				1.05	(1.02–1.09)	0.002						
65–74*social trust				1.09	(0.98–1.20)	0.103						
75 + *social trust				1.03	(0.93–1.13)	0.624						
Age groups*political trust												
35–49*political trust				0.98	(0.93–1.03)	0.393						
50–64*political trust				0.97	(0.01–1.03)	0.325						
65–74*political trust				0.99	(0.92–1.07)	0.877						
75 + *political trust				0.97	(0.90–1.03)	0.382						
Aggregate associational participation							1.01	(1.00–1.03)	0.082	1.01	(0.99–1.03)	0.394
Aggregate social trust							0.83	(0.63–1.09)	0.173	0.96	(0.68–1.38)	0.842
Aggregate political trust							1.38	(1.04–1.85)	0.027	1.43	(1.048–1.97)	0.028
**Cross-level interaction**												
Age groups*aggregate associational engagement												
35–49*associational engagement										1.00	(0.99–1.01)	0.493
50–64*associational engagement										1.01	(1.00–1.02)	0.039
65–74*associational engagement										1.01	(1.00–1.02)	0.168
75 + *associational engagement										1.04	(1.02–1.07)	0.001
Age groups*aggregate social trust												
35–49*social trust										0.88	(0.70–1.11)	0.272
50–64*social trust										0.87	(0.73–1.04)	0.134
65–74*social trust										0.66	(0.53–0.82)	0.000
75 + *social trust										0.60	(0.34–0.83)	0.001
Age groups*aggregate political trust												
35–49*political trust										0.88	(0.80–0.97)	0.014
50–64*political trust										0.94	(0.87–1.02)	0.138
65–74*political trust										1.21	(1.12–1.32)	< .001
75 + *political trust										0.88	(0.69–1.13)	0.325
Constant	0.06	(0.04–0.09)	< .001	0,08	(0.05–0.13)	< .001	0,04	(0.02–0.10)	< .001	0,02	(0.01–0.8)	< .001
Intra-Class Correlation (ICC)	.061568			.0605189			.0485068			.0486823		
AIC	28,438.59			28,411.34			28,438.01			28,383.31		
BIC	28,607.81			28,682.1			23,787.37			28,654.07		
Log-pseudolikelihood	− 14,199.29			− 14,173.67			− 14,196.01			− 14,159.65		
Respondents (countries)	34,935 (33)			34,935 (33)			34,935 (33)			34,935 (33)		

*OR* Odds ratio, *CI* Confidence intervals

In model 2, interaction effects between individual-level social capital variables and different age groups were tested. Higher social trust was associated with higher odds of participating in non-institutionalised activities for individuals in the 50–64 age group (OR 1.08, 95% CI 1.04–1.12). Additionally, associational engagement was associated with higher odds for institutionalised political participation among those aged 75 + (OR 2.15, 95% CI 1.65–2.89). Further, the interaction term for social trust showed that individuals aged 50–64 were more likely to be engaged in institutional political activities (OR 1.05, 95% CI 1.02–1.09).

The findings in model 3 reveal the contextual effects of aggregate levels of social capital on political participation. For non-institutionalised political participation, there was a significant positive association between the aggregate level of associational engagement and the likelihood of being active in political participation (OR 1.04, 95% CI 1.03–1.06). The centred individual-level variable for associational engagement also showed a significant effect (OR 2.03, 95% CI 1.61–2.56), suggesting that both individual and contextual associational engagement are associated with non-institutionalised political participation. For social trust and political trust, the results were more nuanced. The aggregate levels of social trust and political trust did not show a significant association with non-institutionalised political participation, while the centred individual-level social (OR 1.10, 95% CI 1.06–1.14) and political trust were significant (OR 0.92, 95% CI 0.89–0.95). For institutionalised political participation, there was a significant positive association between the aggregate level of political trust and the likelihood of being politically active (OR 1.38, 95% CI 1.04–1.85); however, the association was not significant on the individual level. The aggregate levels of associational engagement and social trust were not significant, while the centred individual-level associational engagement was significantly associated with institutionalised political participation (OR 3.01, 95% CI 2.23-4.06).

**Fig. 1:**
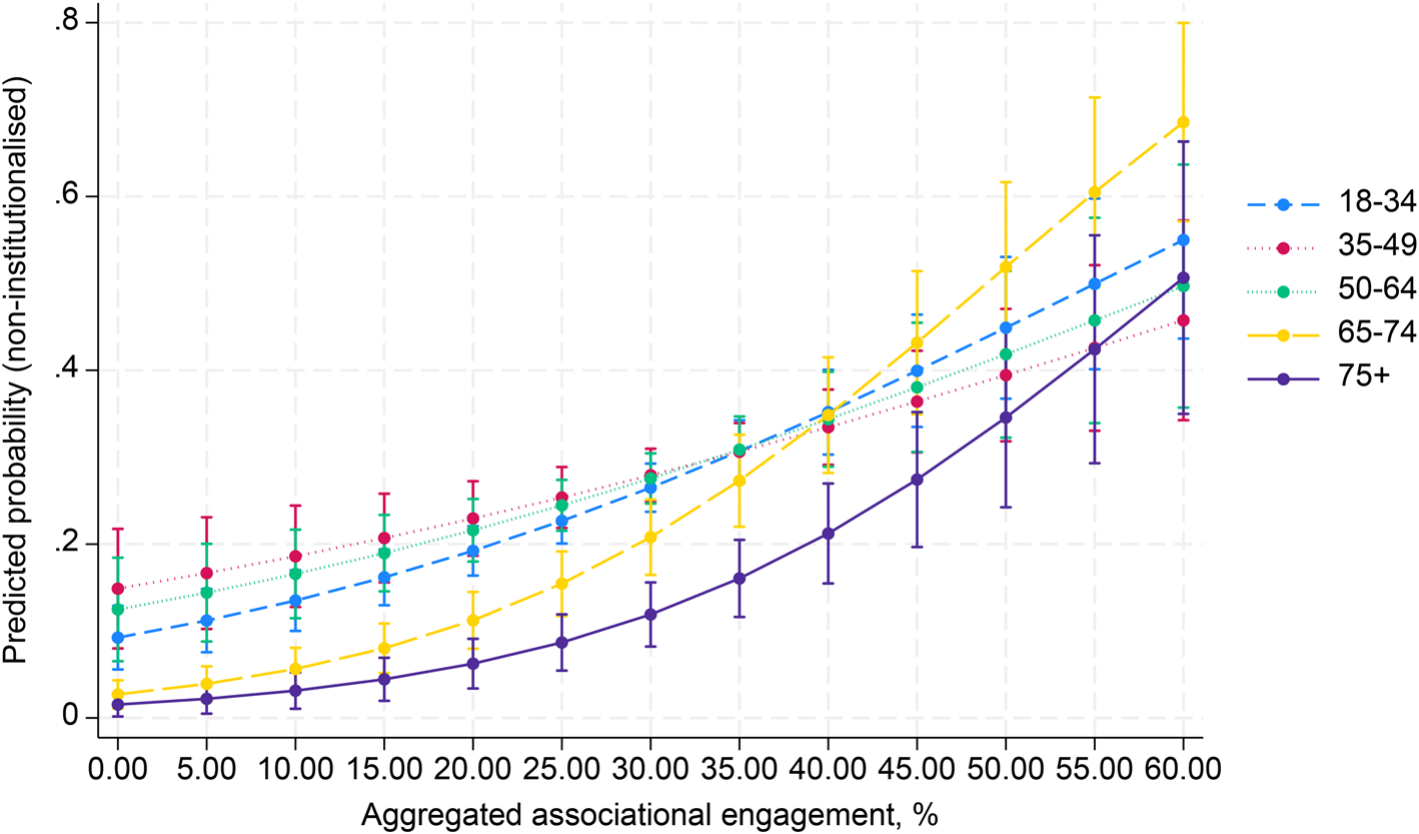
Cross-level interaction with 95% confidence intervals of country-level associational engagement on the association between five age groups and non-institutionalised political participation, given in predicted probabilities, based on model 4, Table 2

**Fig. 2:**
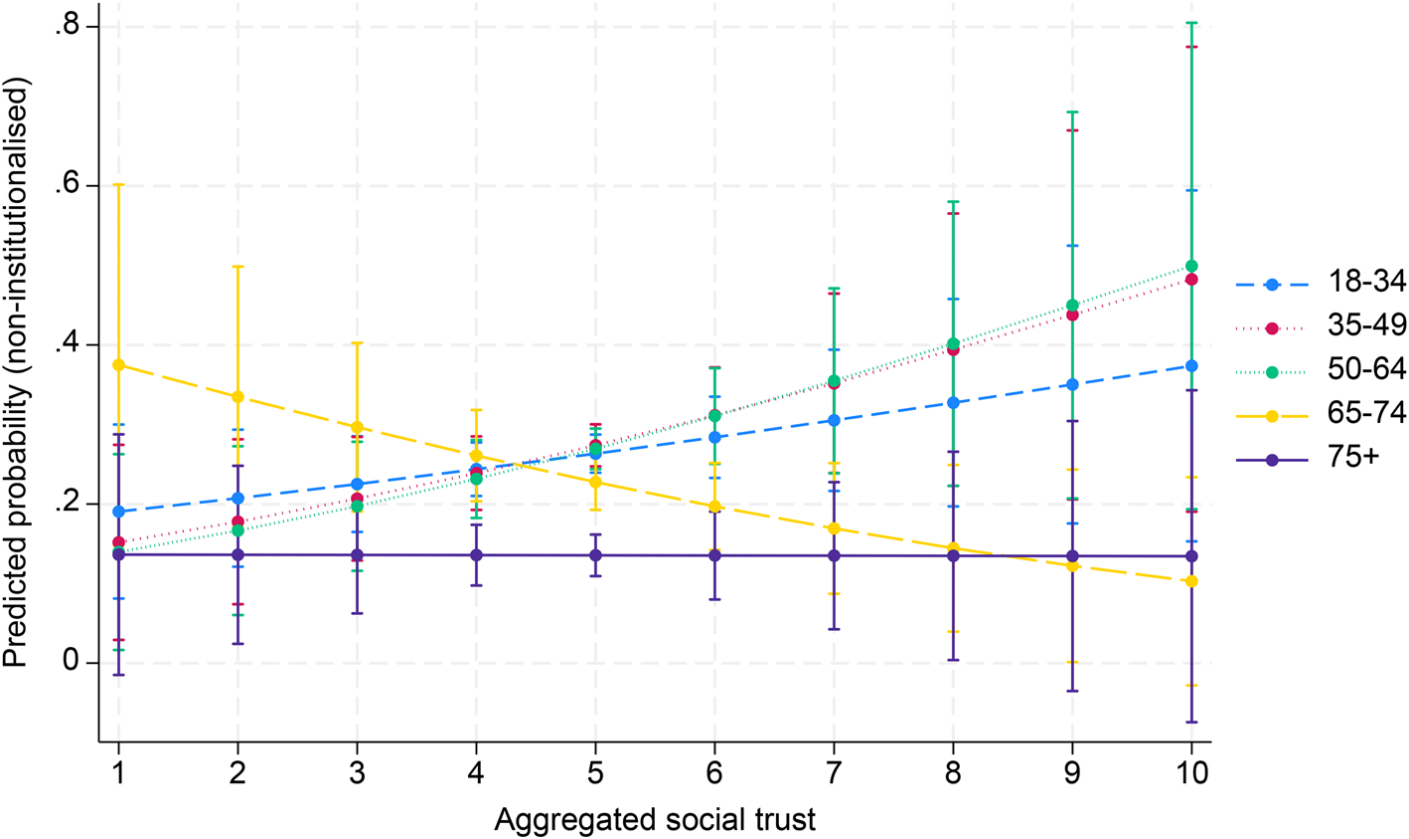
Cross-level interaction with 95% confidence intervals of country-level social trust on the association between five age groups and non-institutionalised political participation, given in predicted probabilities, based on model 4, Table 2

**Fig. 3:**
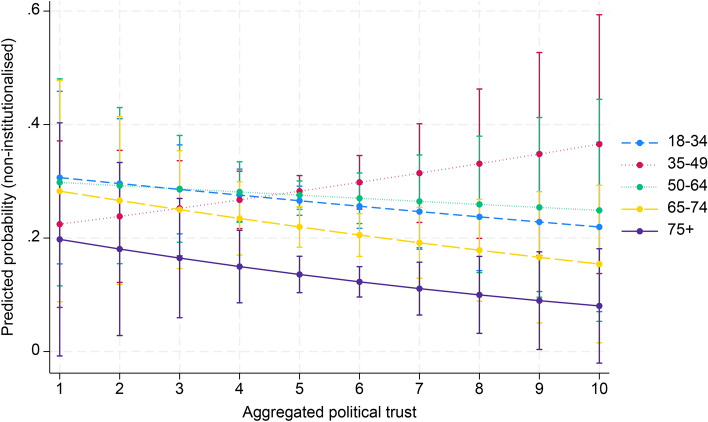
Cross-level interaction with 95% confidence intervals of country-level political trust on the association between five age groups and non-institutionalised political participation, given in predicted probabilities, based on model 4, Table 2

Finally, in model 4 interaction effects were entered to examine whether the effect of aggregated social capital in society moderates the age effect on political participation. Table 2 shows a significant interaction effect between aggregate associational engagement and the 65–74 and 75 + age groups (OR 1.03, 95% CI 1.02–1.05; OR 1.03, 95% CI 1.01–1.05). For the age group 35–49, there was a significant negative association with non-institutionalised political participation (OR 0.98, 95% CI 0.98–0.99). Regarding social trust, a negative interaction was found for the 65–74 age group (OR 0.71, 95% CI 0.52–0.97). In contrast, political trust had a significant positive interaction with the 35–49 age group (OR 1.16, 95% CI 1.06–1.26). Figures 1, 2 and 3 (Appendix 3) illustrate these results. In addition, the marginal effects of aggregated social capital across age groups, presented in Appendix 4, show significant positive associations between country-level associational engagement and non-institutionalised political participation across all age groups, especially for the 65–74 age group (AME = 0.011, SE = 0.001, *p* < 0.001).

**Fig. 4:**
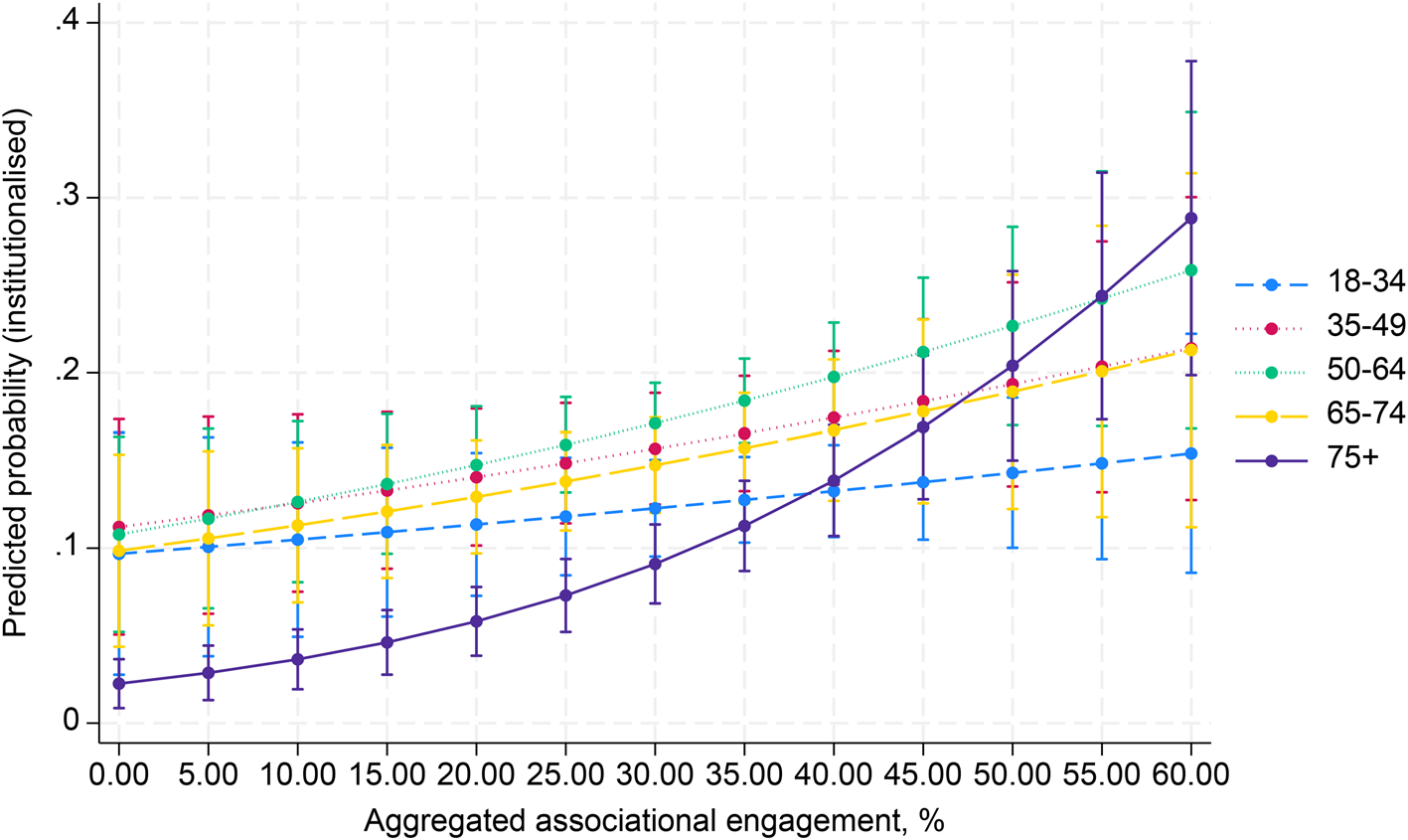
Cross-level interaction with 95% confidence intervals of country-level associational engagement on the association between five age groups and institutionalised political participation, given in predicted probabilities, based on model 4, Table 3

**Fig. 5:**
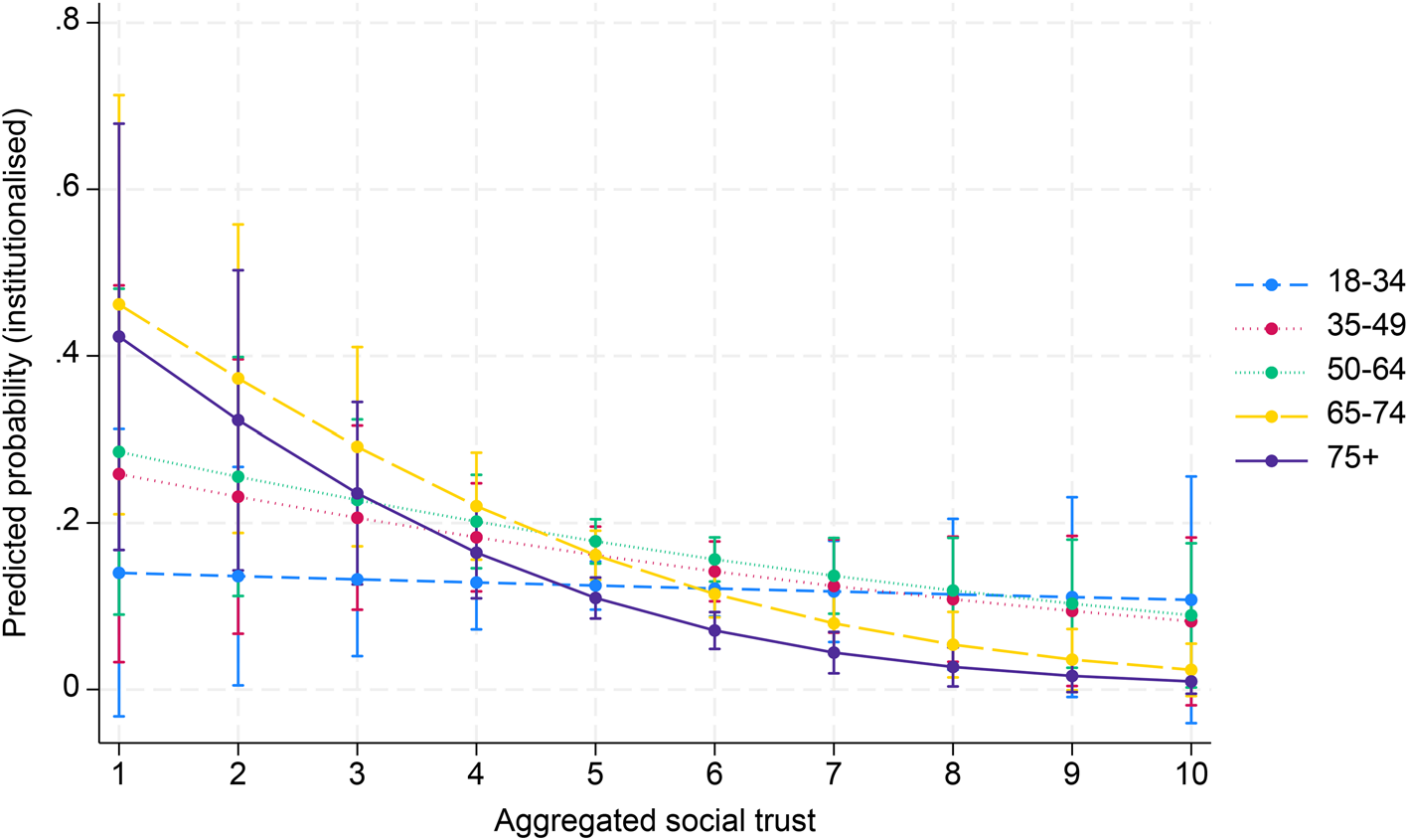
Cross-level interaction with 95% confidence intervals of country-level social trust on the association between five age groups and institutionalised political participation, given in predicted probabilities, based on model 4, Table 3

**Fig. 6:**
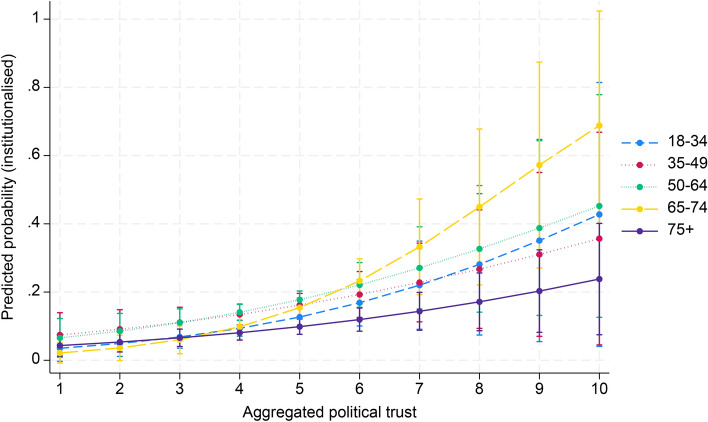
Cross-level interaction with 95% confidence intervals of country-level political trust on the association between five age groups and institutionalised political participation, given in predicted probabilities, based on model 4, Table 3

In Table 3 (model 4), a significant positive interaction effect was observed between aggregate associational engagement and the 75 + (OR 1.04, 95% CI 1.02–1.07) and 50–64 (OR 1.01, 95% CI 1.00–1.02) age groups on institutionalised political participation. Conversely, negative interactions were found between aggregate social trust and the 65–74 (OR 0.66, 95% CI 0.53–0.82) and 75 + (OR 0.60, 95% CI 0.34–0.83) age groups. Political trust had mixed effects, with significant negative interactions in the 35–49 age group (OR 0.88, 95% CI 0.80–0.97) and positive interactions in the 65–74 age group (OR 1.21, 95% CI 1.12–1.32). Figures 4, 5 and 6 (Appendix 3) illustrate these results.

Average marginal effects show that aggregate associational engagement significantly increased institutionalised political participation for the 50–64 (AME = 0.002, SE = 0.001, *p* = 0.032) and the 75 + age groups (AME = 0.004, SE = 0.001, *p* < 0.001). Meanwhile, social trust had significant negative effects on the 65–74 (AME = − 0.051, SE = 0.018, *p* = 0.004) and 75 + (AME = -0.044, SE = 0.014, *p* = 0.001) age groups. Political trust showed a significant positive effect in all age groups, except for the 35–49 age group.

## Discussion

The study’s aim was to assess the importance of social capital on non-institutionalised and institutionalised participation in older adults in comparison with younger age groups in a wide variety of European countries. Age differences were evident in both forms of political participation. When considering broad age categories, younger individuals (18–64 years) were generally more active in political activities, whereas older age groups (65 +) were less likely to participate. However, more detailed regression analyses revealed nuanced patterns within these broad age categories. Specifically, for non-institutionalised political participation, the oldest age group (75 +) was less likely to be active compared to the youngest group (18–34). In contrast, for institutionalised participation, the 65–74 age group was more likely to be active and the 75 + less likely, however, these results were not statistically significant. Further, active associational engagement on an individual level was associated with both forms of political participation, whereas social and political trust were associated with non-institutionalised political participation only, such that individuals with higher social trust and individuals with lower political trust had a higher likelihood of participating. Additionally, we confirmed that political participation is dependent on country characteristics, especially when it comes to non-institutionalised activities. However, of the included aggregated social capital indicators, only country-level associational engagement was associated with non-institutionalised activities and only country-level political trust with institutionalised activities. Finally, the results showed that the association between social capital and non-institutionalised political participation differed between the age groups and that contextual social capital moderated the age effect on both forms of political participation. This moderation included both positive and negative effects depending on the specific age group and type of social capital. For older adults, there was a stronger individual and contextual effect of active associational engagement on political participation, whereas high social trust in the country reduced the likelihood for older adults to be politically active. These main findings will be discussed next.

Our first hypothesis was confirmed in this study: associational engagement appears to enhance both forms of political participation. Our results demonstrate the necessity of acknowledging that the influence of political and social trust may vary when it comes to political participation. This is evident from the findings, which reveal distinct mechanisms for the two forms of participation under scrutiny. Low levels of political trust do not necessarily indicate alienation from the political system as a whole; rather, they imply a preference for non-institutionalised participation, such as demonstrating, signing petitions and boycotting products. The relationship between social trust and political participation has remained elusive in previous research (see e.g. Bäck and Christensen 2016; Levi and Stoker 2000; Uslaner and Brown 2005). According to social capital theory, associational engagement and social trust are intricately intertwined (Putnam 1993). The more an individual trusts, the more likely they are to engage in cooperative civic activities, and vice versa, suggesting a positive association between the two. Not surprisingly, associational engagement and social trust enhance political participation (Nygård and Jakobsson 2013a, 2013b). However, in our study, the relationship was only found to be significant for non-institutionalised political participation. Previous findings (Bäck and Christensen 2016; Marien and Christensen 2013; Uslaner and Brown 2005) support the notion that social trust is more likely to be associated with non-institutionalised political participation. This association might stem from the perception that institutionalised participation can be more time-consuming and tedious (Bäck and Christensen 2016). Additionally, as suggested by Uslaner (2002), social trust tends to have a reduced impact on activities that require long-term commitment and engagement. Based on our findings, institutionalised political participation may require resources beyond social capital for activation. For instance, according to Barrett and Brunton-Smith (2014), diverse psychological factors, including perceptual engagement, attention, affect and motivation, may serve as drivers that influence political participation.

Our second hypothesis was partially supported in our analyses. As expected, the results showed significant variation in both forms of political participation across European countries among older adults (65 +), with values ranging from 0.8 to 54.8% for non-institutionalised participation and from 1.7 to 25.8% for institutionalised political participation. Several country-specific findings regarding political participation among older adults stood out. For instance, the notable involvement of older adults in institutionalised political activities (25.8%) in Turkey presents an intriguing trend that warrants further examination in the future research. Similarly, the diverse age disparities in non-institutionalised political participation observed in countries like Romania raise questions that merit deeper exploration.

Social capital and political participation are culturally sensitive, and the observed variations are likely to reflect differences in political culture, history and the maturity of democracy. For instance, countries with more established and stable democracies, such as Sweden, typically exhibit higher levels of social capital and political participation. In contrast, nations such as Greece, which have experienced more recent transitions and historical legacies, often experience lower levels of participation. Moreover, the varying levels of participation may also reflect other historical characteristics, such as long periods of state control over political life, as is the case for Turkey, with its history of coup d’états, and Serbia, with its communist legacy (Goerres 2009). Tentatively, such factors may influence the level of political participation, such as the high institutionalised participation in Turkey and Serbia.

While we anticipated that country-level characteristics, such as aggregated social capital, might influence political participation, our findings indicate that only associational engagement significantly mattered for non-institutionalised participation. Higher levels of associational engagement within a country suggest increased opportunities for individuals to engage in diverse social, cultural or interest-based groups. This, in turn, can foster a more favourable environment for non-institutionalised political participation. Notably, individual-level social capital retained significance in the models, even after incorporating country-level social capital.

For institutionalised political participation, country-level political trust emerged as a significant positive predictor. This finding highlights that in countries where trust in political institutions is higher, individuals are more likely to engage in institutionalised political activities. This supports the idea that institutional trust fosters engagement within formal political structures by legitimising the political system and encouraging participation (Catterberg and Moreno 2006). However, the individual-level analysis revealed no significant association between political trust and institutionalised political participation, suggesting that while the broader context of political trust matters, individual trust in institutions does not directly translate to higher participation in institutionalised activities.

The significant within and cross-level interaction effects between older adults and associational engagement suggest two key points. First, there was a stronger individual effect of associational engagement for non-institutionalised political participation in the oldest age groups. Second, older adults living in a country with strong associational engagement were also more likely to participate in non-institutionalised political activities. In addition, for the 75 + age group, there was a significant positive effect on institutionalised political participation of living in a country with associational engagement, suggesting that older adults were more likely to be engaged in structured political activities in such environments. These findings support our final hypothesis (H3), suggesting that, especially older adults benefit from living in countries with stronger social capital, at least in the form of associational engagement.

However, significant negative cross-level interactions were observed between social trust and institutionalised political participation among the oldest age groups. This suggests that high social trust in a country might reduce the need or desire for political participation, possibly due to a belief that traditional political institutions are effective and trustworthy. Furthermore, aggregate political trust was positively associated with non-institutionalised political participation for both younger and older age groups. The mixed effects of country-level social and political trust on various forms of political participation across age groups might stem from differing generational attitudes and experiences to political events and policy changes (Catterberg and Moreno 2006), or to variations in social norms, which can differ widely across cultural contexts (Bolzendahl and Coffé 2013). These differences in norms might influence how trust is developed and how it translates into political engagement. This complexity highlights the need for more nuanced policies that recognise social capital as a multifaceted concept, with varying effects depending on the context and demographic factors.

### Limitations

A multilevel approach to studying political participation can contribute to a richer evidence base. However, the way in which non-institutionalised and institutionalised political participation were measured may limit the generalisability of the results. Specifically, the variables etc. The variables used in this study were all dichotomous measures of political participation since the dataset did not include information on intensity/frequency. Thus, we cannot, for instance, rule out that the intensity of the activities engaged in would have given a more nuanced picture of the variation between countries. Additionally, besides social capital, other country-level variables might be expected to influence political participation (Giugni and Grasso 2022). The measure of political trust was included in the definition of social capital (Putnam 2000; OECD 2017); however, there are different perspectives in the literature, with some scholars treating political trust as a distinct construct (e.g. Rothstein and Stolle 2008), separate from broader social capital measures. This variation in conceptualisation may limit the possibilities for comparing results with other studies that either include or exclude political trust as part of social capital. The cross-sectional nature of this study limits our ability to disentangle life cycle and generational effects. Specifically, while the data can assess differences in inferential associations between age, social capital and political participation, it does not allow for the establishment of causal relationships. This limitation means we cannot differentiate between age effects and cohort effects.

Moreover, the data used in this study are from 2016, which may affect its relevance to current trend and contexts. The dataset encompasses only 33 countries, raising concerns about the reliable estimation of country effects, as discussed by Bryan and Jenkins (2016). Finally, an important consideration in our study is the choice of age cut-off for defining the oldest age group. We used 65 years as the cut-off, aligning with the common retirement age in Europe. Therefore, the findings should be interpreted within the context of the chosen age cut-off and the potential implications for generalisability to diverse populations should be considered*.*

## Conclusion

The present study contributes to the literature on social capital and civic engagement in older adults by analysing the less explored aspect of non-institutionalised and institutionalised political participation. Our findings highlight that, while older adults are generally less active in political participation compared to younger age groups, associational engagement remains a critical foundation for political participation in later life. Moreover, high level of social trust in a country was found to reduce the likelihood of political participation among older adults, indicating that trust in established system may discourage active institutional political engagement. The findings suggest that political participation is not solely an individual choice in later life but rather is subject to influences from the societal context.

## Data Availability

The European Quality of Life Survey (EQLS) data is stored at the UK Data Service and is available for research upon request.

## Appendix 1

Dimensions of political participation.

**Table:**

Type of activity	Non-institutionalised political participation	Institutionalised political participation
Attended a protest or demonstration	**0.421**	0.396
Signed a petition	**0.755**	0.118
Commented on a political or social issue online	**0.711**	0.168
Boycotted certain products	**0.729**	0.019
Done voluntary work for political parties, trade unions, social movements or charities	0.360	**0.500**
Contacted a politician or public official	0.141	**0.781**
Attended a meeting of a trade union, a political party or political action group	− 0.042	**0.800**

Note: The table reports factor loadings generated by principal component analysis (varimax rotation with Kaiser normalisation) of the seven items on political participation

Loadings stronger than 0.40 are bolded

## Appendix 2

Multilevel logistic models of testing factors associated with the probability to participate in political participation.

**Table:**

	Non-institutionalised political participation						Institutionalised political participation					
	M0			M1			M0			M1		
	OR	CI 95%	*p* value	OR	CI 95%	*p* value	OR	CI 95%	*p* value	OR	CI	*p* value
Associational engagement				2.16	(1.74–2.70)	< 0.001				3.27	(2.41–4.44)	< 0.001
Social trust				1.11	(1.06–1.16)	< 0.001				1.09	(1.04–1.13)	< 0.001
Political trust				0.97	(0.94–1.01)	0.151				1.05	(1.01–1.09)	0.008
*Age groups* (ref = 18–34)												
35–49				0.91	(0.82–1.01)	0.088				1.30	(1.04–1.64)	0.021
50–64				0.77	(0.67–0.89)	< 0.001				1.34	(1.07–1.68)	0.011
65–74				0.50	(0.37–0.67)	< 0.001				1.05	(0.84–1.31)	0.641
75 +				0.24	(0.17–0.33)	< 0.001				0.59	(0.37–0.94)	0.028
Gender (ref = male)				0.98	(0.88–1.10)	< 0.001				0.64	(0.57–0.73)	< 0.001
*Educational level* (ref = low secondary education or lower)												
Upper secondary or post-secondary				2.23	(1.84–2.69)	< 0.001				1.46	(1.19–1.79)	< 0.001
Tertiary				4.12	(2.93–5.77)	< 0.001				2.50	(1.80–3.47)	< 0.001
Level of urbanisation (ref = the open countryside, a village/small town)				1.33	(1.14–1.56)	< 0.001				1.00	(0.89–1.12)	0.963
*Employment status* (ref = employed)												
Retired				0.49	(0.41–0.58)	< 0.001				0.68	(0.55–0.83)	< 0.001
Other				0.85	(0.77–0.94)	0.002				0.56	(0.52–0.61)	< 0.001
*Marital and cohabiting status* (ref = married or living with partner)												
Divorced or separated				0.97	(0.79–1.20)	0.793				1.02	(0.88–1.19)	0.749
Widowed				0.40	(0.31–0.51)	< 0.001				0.48	(0.43–0.54)	< 0.001
Unmarried				1.46	(1.22–1.73)	< 0.001				0.91	(0.77–1.08)	0.287
Good self-rated health (ref = poor SRH)				1.59	(1.39–1.83)	< 0.001				1.16	(0.95–1.42)	0.143
Ability to make ends meet (ref = with difficulty)				1.12	(1.04–1.20)	0.001				1.36	(0.14–1.61)	< 0.001
Aggregate associational engagement				1.05	(1.04–1.06)	< 0.001				1.03	(1.02–1.04)	< 0.001
Aggregate social trust				1.70	(1.47–1.96)	< 0.001				1.32	(1.12–1.55)	0.001
Aggregate political trust				1.59	(1.30–1.96)	< 0.001				1.43	(1.27–1.61)	< 0.001
Constant	0.310	(0.24–0.40)	< 0.001			0.15	(0.12–0.19)	< 0.001				
Intra Class Correlation (ICC)	0.142						0.09					

M0   Empty model, M1 Bivariate model, CI Confidence intervals

## Appendix 3

See Figs. 1, 2, 3, 4, 5 and 6.

## Appendix 4

See Tables

**Table 4:** Marginal effects of aggregated social capital on non-institutionalised political participation by age group

Age group	AME	SE	*p* value
*Associational engagement*			
18–34	0.007	0.001	< 0.001
35–49	0.005	0.001	0.001
50–64	0.006	0.002	< 0.001
65–74	0.011	0.001	< 0.001
75 +	0.007	0.001	< 0.001
*Social trust*			
18–34	0.020	0.018	0.269
35–49	0.036	0.023	0.124
50–64	0.039	0.024	0.108
65–74	-0.032	0.023	0.158
75 +	0.000	0.020	0.991
*Political trust*			
18–34	− 0.010	0.017	0.575
35–49	0.015	0.020	0.448
50–64	− 0.005	0.022	0.799
65–74	− 0.015	0.020	0.461
75 +	− 0.014	0.019	0.465

*AME* Average marginal effect, *SE* Standard error

4 and

**Table 5:** Average marginal effects of aggregated social capital on institutionalised political participation by age group

Age group	AME	SE	p value
*Associational engagement*			
18–34	0.001	0.001	0.381
35–49	0.002	0.001	0.133
50–64	0.002	0.001	0.032
65–74	0.002	0.001	0.121
75 +	0.004	0.001	< 0.001
*Social trust*			
18–34	− 0.004	0.018	0.842
35–49	− 0.020	0.019	0.289
50–64	− 0.023	0.017	0.179
65–74	− 0.051	0.018	0.004
75 +	− 0.044	0.014	0.001
*Political trust*			
18–34	0.036	0.018	0.041
35–49	0.028	0.018	0.108
50–64	0.039	0.018	0.036
65–74	0.063	0.019	0.001
75 +	0.019	0.008	0.020

*AME* Average marginal effect, *SE* Standard error

5

## References

[CR1] Adler R, Goggin J (2005) What do we mean by ‘“civic engagement”’? J Transform Educ 3:236–253. 10.1177/1541344605276792

[CR2] Bäck M, Christensen HS (2016) When trust matters—a multilevel analysis of the effect of generalized trust on political participation in 25 European democracies. J Civ Soc 12:178–197. 10.1080/17448689.2016.1176730

[CR3] Bäck M (2011) Socialt kapital och politiskt deltagande i Europa [Social capital and political participation in Europe]. Dissertation, Åbo Akademi University

[CR4] Barrett M, Brunton-Smith I (2014) Political and civic engagement and participation: towards an integrative perspective. J Civ Soc 10:5–28. 10.1080/17448689.2013.871911

[CR5] Boerio P, Garavaglia E, Gaia A (2023) Active ageing in Europe: are changes in social capital associated with engagement, initiation and maintenance of activity in later life? Ageing Soc 43:1122–1140. 10.1017/S0144686X21001021

[CR6] Bolzendahl C, Coffé H (2013) Are ‘good’ citizens ‘good’ participants? Testing citizenship norms and political participation across 25 nations. Pol Stud 61:45–65. 10.1111/1467-9248.12010

[CR7] Bryan ML, Jenkins SP (2016) Multilevel modelling of country effects: a cautionary tale. Eur Sociol Rev 32:3–22. 10.1093/esr/jcv059

[CR8] Bui CN, Kim K, Song Q, Jang Y (2023) Political participation among middle-aged and older Asian Americans. Res Aging 45:104–114. 10.1177/01640275221113 35792740 10.1177/01640275221113036

[CR9] Campbell AL (2003) How policies make citizens: senior political activism and the American welfare state. Princeton University Press, Princeton. 10.1515/9781400841318

[CR10] Catterberg G, Moreno A (2006) The individual bases of political trust: trends in new and established democracies. Int J Public Opin R 18:31–48. 10.1093/ijpor/edh081

[CR11] Ekman J, Amnå E (2012) Political participation and civic engagement: towards a new typology. Hum Aff 22:283–300. 10.2478/s13374-012-0024-1

[CR12] Enders CK, Tofighi D (2007) Centering predictor variables in cross-sectional multilevel models: a new look at an old issue. Psychol Methods 12:121–138. 10.1037/1082-989X.12.2.121 17563168 10.1037/1082-989X.12.2.121

[CR13] Eurofound (2018) European quality of life survey 2016: technical and fieldwork report*.* On-line working paper. https://www.eurofound.europa.eu/en/publications/eurofound-paper/2018/european-quality-life-survey-2016-technical-and-fieldwork-report

[CR14] Gabriel OW (2017) Participation and political trust. In: Zmerli S, Meer STWG (eds) Handbook on political trust. Edward Elgar Publishing, Cheltenham, pp 228–241. 10.4337/9781782545118.00025

[CR15] Giugni M, Grasso M (eds) (2022) The Oxford handbook of political participation. Oxford University Press, Oxford

[CR16] Giugni M, Grasso M (2020) Trust, identity, skills, or recruitment?: assessing four explanations of the relationship between associational involvement and the political participation of migrants. Int Mig Rev 54:585–610. 10.1177/01979183198563

[CR17] Goerres A (2009) The political participation of older people in Europe: the greying of our democracies. Palgrave Macmillan, Basingstoke

[CR18] Grasso M, Smith K (2022) Gender inequalities in political participation and political engagement among young people in Europe: are young women less politically engaged than young men? Politics 42:39–57. 10.1177/02633957211028813

[CR19] Hooghe M, Marien S (2012) A comparative analysis of the relation between political trust and forms of political participation in Europe. Eur Soc 15:131–152. 10.1080/14616696.2012.692807

[CR20] Inglehart R, Welzel C (2005) Modernization, cultural change, and democracy: the human development sequence. Cambridge University Press, Cambridge

[CR21] La Due Lake R, Huckfeldt R (1998) Social capital, social networks, and political participation. Polit Psychol 19:567–584. 10.1111/0162-895X.00118

[CR22] Levi M, Stoker L (2000) Political trust and trustworthiness. Annu Rev of Polit Sci 3:475–507

[CR23] Liamputtong P, Rice ZS, Suwankhong D (2022) Social capital and social inclusion. In: Liamputtong P (ed) Handbook of social inclusion. Springer, Cham, pp 43–56. 10.1007/978-3-030-89594-5_3

[CR24] Marien S, Christensen HS (2013) Trust and openness: prerequisites for democratic engagement. In: Demetriou KN (ed) Democracy in transition. Political participation in the European Union. Springer, Heidelberg, pp 109–134

[CR25] Marien S, Hooghe M, Quintelier E (2010) Inequalities in non-institutionalised forms of political participation: a multi-level analysis of 25 countries. Polit Stud 58:187–213. 10.1111/j.1467-9248.2009.0080

[CR26] Mattila M (2020) Does poor health mobilize people into action? Health, political trust, and participation. Eur Polit Sci Rev 12:49–65. 10.1017/S175577391900033X

[CR27] Melo DF, Stockemer D (2014) Age and political participation in Germany, France and the UK: a comparative analysis. Comp Eur Polit 12:33–53. 10.1057/cep.2012.31

[CR28] Norris P (2002) Democratic phoenix: reinventing political activism. Cambridge University Press, Cambridge

[CR29] Nygård M, Jakobsson G (2013a) Political participation of older adults in Scandinavia: the civic voluntarism model revisited? A multi-level analysis of three types of political participation. Int J Ageing Later Life 8:65–96. 10.3384/ijal.1652-8670.12196

[CR30] Nygård M, Jakobsson G (2013b) Senior citizens and political participation—evidence from a Finnish regional study. Ageing Soc 33:159–180. 10.1017/S0144686X1100113

[CR31] Nygård M, Nyqvist F, Steenbeek W, Jakobsson G (2015) Does social capital enhance political participation of older adults? A multi-level analysis of older Finns and Swedes. J Int Comp Soc Policy 31:234–254. 10.1080/21699763.2015.1069207

[CR32] OECD (2017) OECD guidelines on measuring trust. OECD Publishing, Paris. 10.1787/9789264278219-en

[CR33] Pichler F, Wallace C (2007) Patterns of formal and informal social capital in Europe. Eur Sociol Rev 23:423–435. 10.1093/esr/jcm013Purdam

[CR34] Putnam RD (1993) Making democracy work: civic traditions in modern Italy. Princeton University Press, New Jersey

[CR35] Putnam R (2000) Bowling alone: the collapse and revival of American community. Simon and Schuster, New York

[CR36] Rothstein B, Stolle D (2008) The state and social capital: an institutional theory of generalized trust. Comp Polit 40:441–459. 10.5129/001041508X12911362383354

[CR37] Serrat R, Villar F, Celdrán M (2015) Factors associated with Spanish older people’s membership in political organizations: the role of active-aging activities. Eur J Ageing 12:239–247. 10.1007/s10433-015-0341-4 28804357 10.1007/s10433-015-0341-4 PMC5549238

[CR38] Serrat R, Villar F, Giuliani MF, Zacarés JJ (2017) Older people’s participation in political organizations: the role of generativity and its impact on well-being. Educ Gerontol 43:128–138. 10.1080/03601277.2016.1269541

[CR39] Serrat R, Scharf T, Villar F, Gómez C (2020) Fifty-five years of research into older people’s civic participation: recent trends, future directions. Gerontologist 60:e38–e51. 10.1093/geront/gnz021 30889249 10.1093/geront/gnz021 PMC12774630

[CR40] Serrat R, Scharf T, Villar F (2021) Reconceptualising exclusion from civic engagement in later life: Towards a new research agenda. In: Walsh K, Scharf T, Wanka A, Van Regenmortel S (eds) Social exclusion in ageing societies: interdisciplinary and policy perspectives. Springer, Cham, pp 245–257

[CR41] Serrat R, Scharf T, Villar F (2022) Mapping civic engagement in later life: a scoping review of gerontological definitions and a typology proposal. Volunt Int J Volunt Nonprofit Organ 33:615–626. 10.1007/s11266-021-00346-6

[CR42] Serrat R, Nyqvis F, Torres S, Dury S, Näsman M (2023) Civic engagement among foreign-born and native-born older adults living in Europe: a SHARE-based analysis. Eur J Ageing 20:16. 10.1007/s10433-023-00764-z 37166510 10.1007/s10433-023-00764-z PMC10175525

[CR43] Silagadze N, Christensen HS, Sirén R, Grönlund K (2023) Perceptions of inequality and political participation: the moderating role of ideology. Polit Stud Rev 21:285–305. 10.1177/14789299221082037

[CR44] Solevid M, Scheiber Gyllenspetz AI (2022) Capability and political participation among ageing populations. In: Falk Erhag H, Lagerlöf Nilsson U, Rydberg Sterner T, Skoo I (eds) A multidisciplinary approach to capability in age and ageing. International perspectives on aging, vol 31. Springer, Cham, pp 233–248. 10.1007/978-3-030-78063-0_17

[CR45] Tambe EB (2023) Age and political participation in Africa’s electoral regimes. Representation 10(1080/00344893):2173281

[CR46] Taylor KH (2023) Older and still voting? A mixed-methods study of voting amongst the older old in Europe and in the North-West of England. Ageing Soc. 10.1017/S0144686X23000120

[CR47] Teney C, Hanquinet L (2012) High political participation, high social capital? A relational analysis of youth social capital and political participation. Soc Sci Res 41:1213–1226. 10.1016/j.ssresearch.2012.03.012 23017928 10.1016/j.ssresearch.2012.03.012

[CR48] Theocharis Y, van Deth JW (2018) The continuous expansion of citizen participation: a new taxonomy. Eur Polit Sci Rev 10:139–163. 10.1017/S1755773916000230

[CR49] Uslaner EM (2002) The moral foundations of trust. Cambridge University Press, Cambridge

[CR50] Uslaner EM, Brown M (2005) Inequality, trust, and civic engagement. Am Polit Res 33:868–894. 10.1177/1532673X04271903

[CR51] van Deth JW (2015) Political participation. In: Mazzoleni G (ed) The international encyclopedia of political communication. Wiley, West Sussex, pp 1–12

[CR52] Verba S, Schlozman KL, Brady HE (1995) Voice and equality: civic voluntarism in American politics. Harvard University Press, Cambridge

[CR53] Verba S, Schlozman KL, Burns N (2005) Family ties: understanding the intergenerational transmission of political participation. In: Zuckerman A (ed) The social logic of politics: personal networks as contexts for political behavior. Temple University Press, Philadelphia, pp 95–115

[CR54] Weiss J (2020) What is youth political participation? Literature review on youth political participation and political attitudes. Front Polit Sci 2:1. 10.3389/fpos.2020.00001

[CR55] Wenner J, Wagner M (2022) Voting behaviour and health among the oldest-old in Germany: results from a population-based cross-sectional study. J Popul Ageing 16:699–717. 10.1007/s12062-022-09391-5

[CR500] Woolcock M (2001) The place of social capital in understanding social and economic outcomes. Can J Res 2:11-17

[CR56] Zmerli S, Hooghe M (eds) (2011) Political trust: why context matters. ECPR Press, Colchester

